# Infection-Induced Telomere Length Variation: Insights into Pathogenesis of Koala Retrovirus

**DOI:** 10.3390/v17111510

**Published:** 2025-11-17

**Authors:** Hiu Ming Cheung, Sze Wing Jamie Lin, Hanh Thi Hong Nguyen, Tamsyn Stephenson, Natasha Speight, Farhid Hemmatzadeh

**Affiliations:** School of Animal and Veterinary Sciences, The University of Adelaide, Roseworthy Campus, Roseworthy, SA 5371, Australia; hiuming.cheung@adelaide.edu.au (H.M.C.); szewingjamie.lin@student.adelaide.edu.au (S.W.J.L.); nguyenhanhniah@gmail.com (H.T.H.N.); tamsyn.stephenson@adelaide.edu.au (T.S.); natasha.speight@adelaide.edu.au (N.S.)

**Keywords:** koala retrovirus, telomere, telomerase, oncogenesis, cancer, aging

## Abstract

The pathogenesis of koala retrovirus (KoRV) has been explored in various contexts, yet its role in tumorigenesis remains incompletely understood. Unlike acute transforming retroviruses, KoRV lacks a viral oncogene but may contribute to oncogenesis via indirect mechanisms. However, the relationship between KoRV and telomere length, as a potential indicator of telomerase activity, has not been examined. This study investigates the effect of KoRV infection on telomere length in 47 samples from Southern Australian koalas in a novel telomere length quantification method. Telomere lengths of 30 KoRV-negative samples were compared to those of 17 KoRV-positive samples using the Absolute Human Telomere Length Quantification qPCR kit (ScienCell Research Laboratories, California, USA). The telomere length in KoRV-infected WBCs was significantly longer than the uninfected ones (*t* = −2.059, *p*-value = 0.045). In line with this, telomere length correlated positively with proviral load (*r* = 0.421, *p*-value = 0.003), further linking viral burden to telomere elongation. Furthermore, the effect of age on telomere length differed by infection status (*β* = −5329.7, *p*-value = 0.0038); KoRV-positive individuals exhibited longer telomeres at a younger age but experienced more rapid telomere attrition over time compared to KoRV-negative individuals. These results suggest KoRV promotes telomerase elongation ability and modulates age-related telomere dynamics, potentially contributing to subsequent cellular immortality and oncogenesis. These pathways may overlap with other retroviruses, where telomerase dysregulation contributes to their oncogenic potential. This study provides new insights into KoRV pathogenesis and DNA quantification methodology, which could be valuable for future research by identifying predictive markers for tumour progression and potential therapeutic targets in affected koalas.

## 1. Introduction

Koala retrovirus (KoRV) is a critical pathogen threatening the survival of koalas (*Phascolarctos cinereus*), an iconic Australian marsupial now classified as endangered by the Australian government due to habitat loss, climate crisis, and diseases. KoRV is believed to be an important contributor to certain cancers, including leukemia, lymphoma, and may also exacerbate clinical chlamydiosis in koalas through immunosuppression [1]. KoRV infection rates differ across regions, with 100% prevalence in the Northern Australian population (Queensland and New South Wales) and a lower prevalence in the Southern population (48% in South Australia and 30% in Victoria) [2,3].

Koalas exhibit a strikingly higher rate of neoplasms than humans [4]. This heightened susceptibility has been positively correlated with the presence of KoRV and its proviral DNA [1]. Koala retrovirus belongs to the Retroviridae family that is known to induce oncogenic transformation via various mechanisms. One of these is where proviral integration directly activates proto-oncogenes; Retroviruses lacking oncogenes can alter the expression of host genes through insertional mutagenesis, providing new transcriptional regulatory sequences. While KoRV is not known to contain a viral oncogene, recent studies found that the integration sites of KoRV cluster near known cancer genes, and the mutational burden of endogenization increases the frequency of neoplasia [4]. Upregulation of several oncogenes was also discovered when a human cell line was infected with KoRV [5]. The establishment of the Koala Genome Consortium and the publication of the first fully assembled koala genome will also provide further deeper understanding of KoRV genetics, including viral subtypes, recombinants, and preferences for viral integration [6].

In other species, it has been found that retroviral oncogenic potential was linked to telomerase activity dysregulation. For example, bovine leukemia virus (BLV), human immunodeficiency virus (HIV-1), human T-lymphotropic viruses (HTLV-I and HTLV-II), and avian leukosis virus subgroup J (ALV-J) have all been shown to alter telomerase activities and to cause cancers [7,8,9]. Human Immunodeficiency Virus (HIV-1) infection is known to decrease telomerase activity in CD4+ T cells. This decrease in telomerase activity was thought to limit the proliferation of this immune cell and to contribute to the immunodeficiency observed in infected individuals [10]. Human T-lymphotropic viruses (HTLV-I and HTLV-II) are two closely related retroviruses that cause T-cell leukemia/lymphoma in humans. In HTLV-I, the transactivator protein (Tax) induces telomere reverse transcriptase gene expression via the NF-κB signaling pathway, leading to increased telomerase activity [11]. HTLV-II, on the other hand, increases telomerase activity in uninfected CD34+ progenitor cells through a synergistic interaction with *Bcl-2* [12]. Avian Leukosis Virus Subgroup J (ALV-J) infection has been shown to cause lymphomas in chickens through concurrent dysregulation of *c-Myc* and telomerase, which functionally cooperate to promote malignant transformation [8]. Bovine Leukemia Virus (BLV) infection has been associated with increased telomerase activity in infected lymphocytes and contributes to the development of leukemia in infected animals [7]. Therefore, the ability to control telomerase activity could be a common characteristic of many retroviruses, despite their diverse mechanisms of cancer induction. This telomere dysregulation contributes to the development of various diseases and serves as an alternative pathway in oncogenesis.

Cells have built-in limits to how often they can divide. This is because telomeres, the protective cap at the ends of chromosomes, shorten in each cell cycle due to incomplete DNA replication. Different species have varying rates of telomere shortening [13]. Eventually, critically short telomeres will trigger apoptosis [13,14]. Telomerase enzyme counteracts this erosion by adding sequences of TTAGGGG telomeric repeats back to the telomeres, mostly by its catalytic subunit telomerase reverse transcriptase (TERT) using an internal RNA template known as telomerase RNA component (TERC), thereby extending cell lifespan [14,15,16]. Telomerase is active primarily in lymphocytes, stem cells, and germ cells. However, cancer cells often exhibit upregulated telomerase activity, which allows them to divide indefinitely and attain immortality [16,17,18]. It can be used as a hallmark of cancer detection as it is present in 90% of human tumours [19,20]. Furthermore, another study analysed 31 different cancers in the Cancer Genome Atlas patients’ cohort and found that 73% of the cases had increased telomerase expression and activities, which were associated with point mutation, rearrangement, and amplification in the TERT promoter [21]. In an experimental trial in mice, the TERT mutant promoter not only produced poorly differentiated thyroid tumours, but also overactivated cytokines and chemokines signalling pathways, which is not normally seen in those tumours lacking the TERT mutant [22].

Therefore, telomerase dysregulation of both retroviral-infected cells and cancers raises the question of whether koala retrovirus also similarly disrupts telomerase activity as an oncogenic mechanism. Despite the current advances in understanding of pathogenesis in koala retrovirus, this potential mechanism has not yet been fully investigated until now.

Our study evaluates the telomere length in KoRV-positive and -negative samples, accounting for age as a covariate, with the hypothesis that an increased telomere length would be found in infected koalas. If so, elevated telomerase activity in retrovirus-infected cells may serve as a predictive marker for tumour progression and therapeutic targets [23,24]. Additionally, this investigation may lead to further studies of telomerase activities in other tumour-inducing retroviruses in animals and humans.

## 2. Materials and Methods

### 2.1. Samples

KoRV-positive and -negative koala samples were sourced from a previous study of KoRV in the population of Mount Lofty Ranges, South Australia [25]. All koalas were euthanised by veterinarians on welfare grounds and received a full necropsy at the Veterinary Diagnostic Laboratory, Roseworthy Campus, University of Adelaide. Blood samples were taken at euthanasia and were stored in EDTA at −20 °C prior to DNA analysis. DNA was extracted from whole blood using the QIAMP DNeasy Minikit (Qiagen, Hilgen, Germany) and stored at −20 °C. After the collection of blood, leukocyte counts were available for 9 of the samples (range from 0.232 to 7.19 10^9^/L).

KoRV status of the samples was determined based on the presence or absence of the following genes, namely *gag 2*, *pol*, *env 1*, and *env 2*, via conventional PCR. Samples were classified as positive if all four genes were detected and negative if *pol* and the other genes were not detected. This method is consistent with previously described approaches used to distinguish intact exogenous “pol-positive” KoRV infection from fragmented KoRV sequences that are present in southern koalas [26]. The proviral load was quantified by qPCR targeting the KoRV *pol* gene.

A total of 52 DNA samples were available, comprising 18 KoRV-positive and 34 KoRV-negative koalas, ranging in age between tooth wear classes I to VI [27]. There were no instances of neoplasia or lymphoma detected in any of the koalas, based on necropsy findings, lymph node histology, and leukocyte counts [25].

The sampling was approved by the University of Adelaide Animal Ethics committee (Approval number: S-2016-169) and SA Government Department of Environment, Water and Natural Resources (Scientific Purpose Permit Y26054) and conducted in accordance with the guidelines set out in the ‘Australian Code for the care and use of animals for scientific purposes 8th edition (2013)’ by National Health and Medical Research Council, Canberra.

### 2.2. Quantitative PCR for Telomere Length Quantification

All extracted DNA was quantified using a NanoDrop spectrophotometer (Thermo Fisher Scientific Inc., Wilmington, DE, USA) and stored at −20 °C. The Absolute Human Telomere Length Quantification (AHTLQ) qPCR Assay Kit (ScienCell Research Laboratories, Carlsbad, CA, USA) was used to directly measure the cycle threshold (*Ct*) values, reflecting the average telomere length in white blood cells. The TEL primer set was used to recognise and amplify the telomere sequences. The SCR primer set was used to recognise and amplify a 100 bp-long region on human chromosome 17 and serves as a reference for data normalization. 200 µL of nuclease-free water was added to each primer set to make the TEL primer stock solution and SCR primer stock solution. Human genomic DNA with a telomere length of 1.23 ± 0.09 Mb per diploid cell was used as the reference genomic DNA sample. The 7 μL qPCR reactions were dispensed using an epMotion 5075 liquid handler (Eppendorf, Hamburg, Germany). There were 2 reactions per DNA sample using 2 different primer sets. Each reaction consisted of 0.35 μL reference genomic DNA sample or collected DNA sample, 0.7 μL of TEL or SCR primer stock solution, 3.5 μL of 2X GoldNStart TaqGreen qPCR master mix, and 2.45 μL of nuclease-free water. All reactions were run in three replicates.

The qPCR plate was centrifuged at 1500× *g* for 15 s and run in QuantStudio^TM^ Real-Time PCR System (Thermo Fisher Scientific Inc., Wilmington, DE, USA). The temperature and timing of the qPCR reactions were initial denaturation at 95 °C for 10 min; 32 cycles of denaturation at 95 °C for 20 s; annealing at 52 °C for 20 s; and extension at 72 °C for 45 s.

The qPCR data were recorded and analysed in QuantStudio^TM^ Real-Time PCR Software v1.7.2 (Thermo Fisher Scientific Inc., Wilmington, DE, USA). The obtained *Ct* values were used to calculate the telomere length of the sample, where the sample telomere length in Mb equals 2^(−(differences in Ct values between ΔTEL and ΔSCR))×1.23)32, as per the manufacturer’s instructions. ΔTEL and ΔSCR refer to the difference in *Ct* values between the samples and the human reference of primer TEL and SCR, respectively. The number of chromosomes in a koala diploid cell is 32. This *Ct* [2^−ΔΔ*Ct*^] comparative method was used to calculate the relative expression levels of each amplicon.

### 2.3. Statistical Analysis

To compare the telomere length between infection-positive and -negative groups, an independent *t*-test was performed. The association between proviral load and telomere length was assessed using both Pearson’s correlation and simple linear regression. Pearson’s correlation was used to test the direction and strength of the association (reported as the *r* and the *p*-value). Simple linear regression was used to visualise the trend, estimate the regression line with a 95% confidence interval, and determine the *R*-squared value. To control for the potential confounding effect of age, telomere length was considered as an age-related factor in relation to KoRV infection status. An interaction term was included in a linear model to test whether the association between infection and telomere length varied with age. Other factors, including DNA concentration and leukocyte count, were also taken into account in the data analysis. Descriptive statistics, group comparisons, and Pearson’s correlation were performed using the SPSS Statistics software v26 (IBM, NY, USA). Linear modelling, including simple linear regression of proviral load versus telomere length, and the interaction term between age and KoRV status, was conducted in R statistical software (Version 4.4.2, R Core Team, Vienna, Austria). The level of statistical significance was set at *p*-Value = 0.05.

## 3. Results

### 3.1. Average Telomere Length

A total of 52 blood samples were available from wild koalas in the previous study (18 KoRV-positive and 34 KoRV-negative). Due to the DNA quality and quantity, 47 DNA samples were included in this analysis (Table 1). Five samples (1 KoRV-positive and 4 KoRV-negative, 9.6%) were eliminated from the study; two KoRV-negative samples due to DNA degradation, two *Ct* values were greater than 28 and considered as false positives according to the manufacturer’s instructions, and one outlier in KoRV-positive samples due to exceeding plausible biological ranges of DNA length (179,602 Kb; *Ct*_(SCR)_ 27.628).

**Figure 1:**
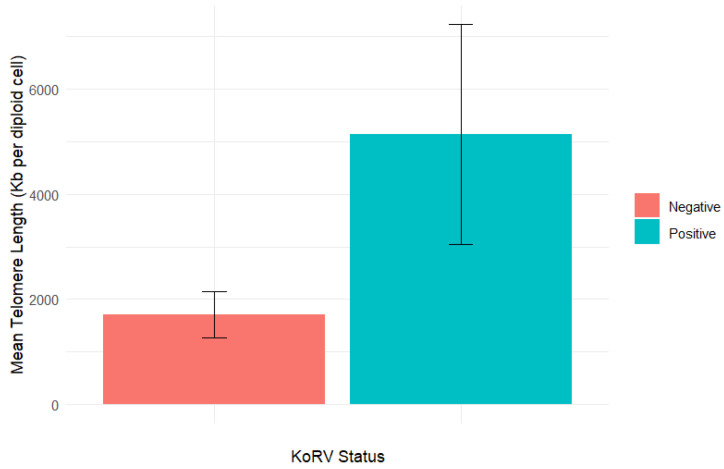
Comparison of telomere length in KoRV-infected (*n* = 17) and non-infected (*n* = 30) koalas. Infected animals showed a significantly higher telomere length based on the independent *t*-test (*t* = −2.059; *p*-value = 0.045). Error bars represent standard errors.

The koala samples showed a typical, clean, sigmoidal qPCR amplification curve (Figures S1 and S2). Overall, infected koalas showed significantly higher telomere length (Mean+/-SD = 5140 ± 8622 vs. 1707 ± 2399; *t* = −2.059, *p*-Value = 0.045) compared to KoRV-negative counterparts (Figure 1).

### 3.2. Proviral-Load Analysis

**Figure 2:**
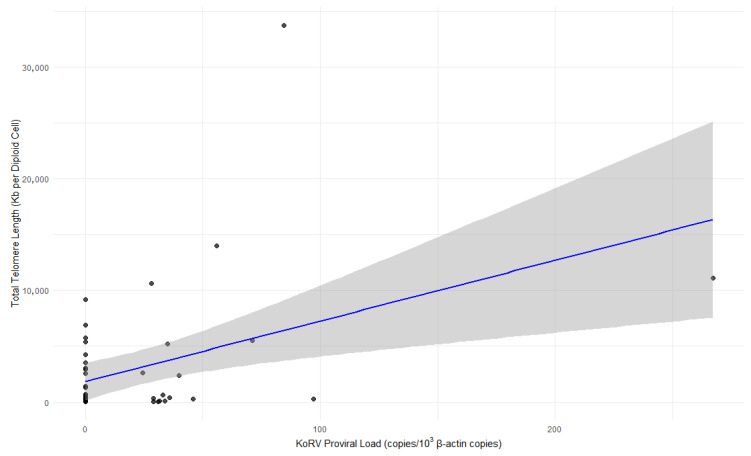
Scatter plot showing the relationship between KoRV proviral load (copies per 10^3^ β-actin copies) and total telomere length (Kb per diploid cell). Each point represents an individual sample. The solid line represents the regression estimate, and the shaded area represents the 95% confidence interval (CI) for the mean regression line (*R*^2^ = 0.178). A significant positive correlation between the two variables was also detected by Pearson’s correlation analysis (*r* = 0.421, *p*-value = 0.003).

Samples (*n* = 47) were also analysed for their relationship between proviral load and telomere lengths. A significant positive correlation was found (*r* = 0.421, *p*-value = 0.003), suggesting that higher proviral load is associated with longer telomere lengths (Figure 2). The linear regression yielded an *R*-squared value of 0.178.

### 3.3. Interaction Analysis of Age and Infection Status

**Figure 3:**
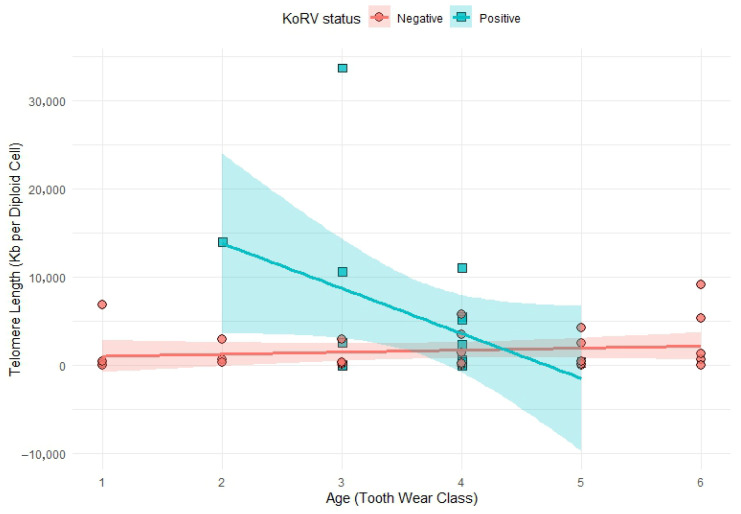
Scatter plot showing the relationship between age and total telomere length (Kb per diploid cell) with different KoRV infection status. Each point represents an individual sample. The solid lines represent the regression estimate, and the shaded areas represent the 95% confidence interval (CI) for the mean regression line. Negative values are shown as a result of the regression model’s extrapolation, due to sparse sampling at age extremes.

To investigate the impact of age on telomere length, we fitted a linear model with an interaction term between age (Tooth Wear Class) and KoRV status. The interaction was statistically significant (*β* = −5329.7, *p*-value = 0.0038), indicating the effect of age on telomere length differs between KoRV-positive and KoRV-negative individuals. Telomere length declined more rapidly with age in KoRV-positive individuals, while it remained relatively stable or slightly increasing in KoRV-negative individuals (Figure 3). Notably, KoRV-positive animals tended to have longer telomeres at a younger age, but this difference diminished with increasing age, with a trend toward longer telomeres in KoRV-negative individuals in older age classes. However, the interpretation of this early-life pattern is limited by a small sample size (*n* = 1) of young KoRV-positive individuals.

## 4. Discussion

The pathogenesis and clinical features of KoRV are still being investigated. However, existing research suggests that KoRV can cause neoplastic conditions, such as lymphoma and leukemia, as well as immunosuppression and predisposition to chlamydiosis [1,2,28,29]. Increased proviral loads have also been found in koalas with neoplasia [30]. Given these associations, it is noteworthy that our current study found both a significantly higher telomere length in KoRV-infected koalas compared with those that were KoRV-negative and a significant positive correlation between proviral load and telomere length. The mechanisms may be similar to other retroviral infections in vertebrate species [31]. For example, BLV-associated neoplasia has been attributed to viral interaction with telomerase, which inhibits telomere shortening [7]. Therefore, it was hypothesised that telomerase activity may play an important role in KoRV tumorigenesis. Although no studies have investigated the relationship between KoRV and telomerase dysregulation, the observed association between higher proviral load and longer telomeres suggests a potential mechanistic link.

Telomere shortening is a natural process that occurs in somatic cells and contributes to cell senescence and cell death by limiting cell proliferation [14]. Cellular senescence can also be triggered by other stressors such as oxidative damage, DNA damage, chromatin changes, and overexpression of oncogenes [32]. However, the induction of cell senescence can be counteracted through carcinogen- or virus-mediated transformation [14]. This is because, although cancer cells have been shown to have shorter telomeres than their surrounding non-transformed cells [18,23], activation of telomerases is involved in tumorigenesis by synthesising telomeric repeat DNA at the end of chromosomes to stabilise the telomeres [18]. Therefore, with the help of telomerases, unlimited and uncontrolled cell proliferation can be achieved in cancer cells, leading to cell immortality [33,34,35]. Hence, observations of elevated values of telomerase activity in both human tumour cell lines and BLV-infected cattle support the hypothesis that telomerase is responsible for cell proliferation and retroviral oncogenesis [23].

Recent studies have provided novel insights into the oncogenesis of KoRV. Hub genes involved in regulating the cell cycle were found to be more highly expressed in healthy koalas compared to those infected with KoRV [36]. Additionally, transcriptional dysregulation in cancer and viral carcinogenesis pathways were found to be upregulated in infected individuals [5,36]. In human cells experimentally infected with KoRV, multiple oncogenes were upregulated, while miRNAs involved in immune response and apoptosis were downregulated [5]. Further studies have identified specific oncogenic mechanisms associated with KoRV infection, including preferential integration near known host oncogenes such as *Bcl-2* and *myc*, suggesting that insertional mutagenesis may contribute to their dysregulation [4]. In KoRV-infected koalas with neoplasia, *Bcl-2* expression was significantly upregulated, particularly in those infected with multiple KoRV subtypes [37]. Similarly, *myc* was a frequent target of KoRV proviral insertion [4] and found to be upregulated in tumour-bearing as well as subclinically infected animals [37]. These findings suggest that early dysregulation of *myc* may precede neoplastic transformation, and that cooperative mechanisms such as concurrent *Bcl-2* upregulation may be required for full malignant transformation [37].

Also, genes associated with telomere organisation were found to be upregulated in KoRV-infected cells [5], suggesting KoRV may interrupt telomere maintenance alongside oncogene activation. Taken together with our findings, these studies support a model in which oncogene regulation and telomerase activity jointly contribute to tumorigenesis in KoRV-infected koalas. The primary hallmarks of tumour cells include uncontrolled growth, dysplasia, and anaplasia, leading to cellular immortality and malignancy [38]. Both oncogene activation and telomerase expression are essential drivers of tumour development [38]. Oncogenes promote uncontrolled cell proliferation by overriding the normal growth-regulatory mechanism, while telomerase prevents and bypasses cell senescence, thereby sustaining proliferation without aberrant growth control [32,34,38]. These mechanisms closely resemble those described in other retroviruses, such as HTLV-II and ALV-J, where synergistic activation of telomerase, anti-apoptotic pathways, and proliferative oncogenes drives malignant transformation [8,12]. While there are still many details yet to be discovered and understood, these findings provide a foundation for further mechanistic studies into KoRV oncogenic potential, particularly in the context of pathways shared with other retroviruses.

In this study, telomere length exhibited considerable inter-individual variability, which is expected in wild populations where multiple biological and environmental factors influence telomere dynamics, including age, antigenic stimulation of the resting lymphocytes, different organ sampling sites, and genetic variations [7,39]. To account for this natural variation, we analysed the effects of age together with KoRV infection status by using an interaction term in the linear model, which tested whether telomere attrition with age differs according to KoRV infection status. The significant interaction observed supports that KoRV infection modulates telomere dynamics across the lifespan. KoRV-positive individuals had longer telomeres at a younger age, consistent with our hypothesis and previous reports of elevated telomerase activities in early life, including in other retroviral infections such as BLV [7,16,24]. However, telomere length in KoRV-positive individuals declined more rapidly with age than in uninfected counterparts. This suggests that KoRV infection may accelerate age-related telomere attrition, potentially reflecting virus-induced cellular aging, cumulative immune activation, or immune exhaustion. We also observed residual within-group variation, particularly among KoRV-negative koalas. This may reflect individual differences in the factors mentioned above, such as genetic background or exposure to other infectious stressors [7,39], or technical or biological factors specific to koala telomere architecture. For instance, koalas possess discontinuous telomeric repeats [40], which may increase variability in qPCR-based telomere estimates. Nevertheless, all reactions displayed clean amplification profiles with consistent efficiency across samples, suggesting minimal technical error.

Given the influence of age on telomere dynamics, the within-group variation found in this study may reflect age-dependent changes in telomere maintenance mechanisms. As telomeres shorten in each cell division, older individuals have shorter telomeres and fewer cell divisions than younger individuals [41,42]. Loss of telomeres not only induces antiproliferative signals leading to cellular senescence, but also contributes to the decline in tissue function, integrity, performance, and reserve capacity, which are hallmarks of ageing [32,42]. As organisms age, senescent cells accumulate, promoting growth and neoplastic progression of mutant cells, and also reducing immune function due to restructuring processes [42,43]. For koalas that are horizontally infected early in life, the presence of viral sequence may modulate telomerase activity during the immunologically naïve stage, before significant telomere erosion has progressed. This could temporarily delay attrition initially and create an early-life telomere advantage. However, this initial advantage may come at a cost, with accelerated shortening later in life due to cumulative stress and immune activation. Therefore, this dual-phase pattern, initial elongation followed by accelerated decline, suggests that the impact of retroviral infection on telomere dynamics may be age-dependent. Our findings highlight the importance of considering age when interpreting telomere length in KoRV-affected populations, and may reflect differential effects on cellular senescence or immune activation at different life stages.

Telomere length represents a combined outcome of cumulative proliferation history, telomerase activity, and clonal selection [14,44], which are themselves shaped by organism-level factors such as age, genetic background, and antigenic stimulation. Consequently, telomerase upregulation may not instantaneously correspond to an increased mean telomere length [7,39]. Comparable studies in other retroviral systems (e.g., BLV) have reported an elevated telomerase activity in hosts while telomere length differences remained variable or non-significant [23], reflecting the complexity of telomere regulation in vivo. Our findings of increased mean telomere length in KoRV-positive koala blood cells, together with a positive association with proviral load, are consistent with KoRV-associated modulation of telomere dynamics, potentially through effects on lymphocytic proliferation or telomerase activation. Thus, while telomere length reflects a cumulative record of telomerase activity rather than an instantaneous measure, it provides a biologically meaningful, population-level indicator of altered telomere maintenance.

The mechanisms of KoRV infection are likely to be complex. While transcriptomic studies have revealed upregulation of genes involved in telomere organisation, no specific candidate gene has yet been identified that directly links telomerase activity and the infection process. It remains worthwhile to explore whether key telomere-regulating genes, including those modulating telomerase expression, and telomeric binding proteins, are differentially expressed and functionally altered in these individuals. Genes of interest, such as *Myc*, *Bcl-2*, *Myb*, and *Flt3*, are implicated in the oncogenic processes of other retroviruses [8,12,45,46] and warrant further investigation. This may provide valuable insights into the molecular interplay between KoRV and telomere maintenance pathways, as several of these genes are known to influence telomerase activity [47,48,49]. In parallel, direct telomerase assays could help determine whether KoRV infection leads to functional upregulation of telomerase, complementing transcriptomic and gene expression findings. Moreover, since the target cells of KoRV are lymphocytes [50], a cell type known to exhibit telomerase activity similar to that observed in tumour cells [51], targeted lymph node expression studies could be a promising area for further research in understanding the virus’s role in immunosuppression and neoplasia induction. While the number of koala samples were sufficient in this study, larger sample sizes may be needed to validate the statistical significance of the observed associations and to more precisely characterise the individual-level variation in telomere dynamics. Future studies may benefit from targeted sampling of KoRV-positive individuals in Northern populations where infection prevalence is higher, to better characterise telomere dynamics across the full age spectrum. Additionally, captive populations with a known family history of neoplasia may serve as ideal cohorts for serial blood collection or longitudinal studies tracking telomere length and expression of candidate genes in both KoRV-positive and KoRV-negative individuals. Such studies may help identify early predictive markers of tumourigenesis and determine whether early telomere advantage predicts future tumour risks.

## 5. Conclusions

This study found a significant increase in telomere length in KoRV-positive koalas, resulting from telomerase activity upregulation. These findings could provide valuable insights into an alternative pathogenic pathway of KoRV. Not only could this help in understanding how the virus contributes to neoplasia development through the interaction with telomerase activity and cell proliferation, but it also raises the possibility of using telomerase inhibition therapy, known for its effectiveness in treating tumours [42]. Novel use of a human AHTLQ test kit in koalas was successful, despite their unusual telomere structure [40]. This also suggests the assay’s cross-species applicability and offers an alternative approach for telomere studies in other non-human species. Overall, further investigation is still required to unravel all the remaining unanswered questions about KoRV.

## Figures and Tables

**Table 1:** Telomere length based on KoRV infection status and tooth wear class of koalas.

KoRV Positive				KoRV Negative			
Tooth Wear Class	*Ct* (SCR)	*Ct* (TEL)	Telomere Length (Kb)	Tooth Wear Class	*Ct* (SCR)	*Ct* (TEL)	Telomere Length (Kb)
				I	19.769	11.619	72
				I	20.608	10.508	278
				I	21.152	10.201	501
				I	26.382	11.645	6906
II	27.428	11.675	13,963	II	21.004	11.249	370
				II	22.036	11.515	724
				II	25.059	10.188	3020
III	17.548	11.387	18	III	19.047	10.460	97
III	19.809	11.249	95	III	20.888	10.93	252
III	24.334	11.007	2599	III	20.790	10.467	324
III	24.994	9.639	10,595	III	23.065	10.188	2919
III	25.810	9.403	33,723				
IV	17.601	11.645	16	IV	17.827	11.475	21
IV	19.681	10.873	113	IV	19.520	10.807	106
IV	21.953	11.730	302	IV	18.156	9.558	150
IV	21.095	10.583	369	IV	20.679	11.097	194
IV	22.135	10.797	655	IV	20.414	10.427	257
IV	24.499	11.280	2411	IV	20.723	10.599	282
IV	27.212	12.874	5236	IV	23.394	10.950	1409
IV	26.670	12.259	5508	IV	25.404	11.631	3540
IV	25.917	11.115	11,086	IV	23.135	9.281	5746
V	21.312	11.060	308	V	19.323	11.546	56
V	22.139	11.604	375	V	18.825	10.119	106
				V	21.508	10.640	472
				V	24.275	10.981	2540
				V	25.428	11.386	4267
				VI	18.417	10.473	62
				VI	22.564	11.131	699
				VI	23.357	11.031	1298
				VI	26.773	12.398	5371
				VI	27.133	11.986	9174

## Data Availability

The original contributions presented in this study are included in the article/Supplementary Material. Further inquiries can be directed to the corresponding author(s).

## Supplementary Materials

The following supporting information can be downloaded at: https://www.mdpi.com/article/10.3390/v17111510/s1. Figure S1: Amplification plot showing successful qPCR amplification upon TEL and SCR primers in the first batch of the experiment containing 12 samples, including positive and negative controls; Figure S2: amplification plot showing successful qPCR amplification upon TEL and SCR primers in the second batch of the experiment containing 50 samples, including positive and negative controls.
