# Supplementary material for: Infection-Induced Telomere Length Variation: Insights into Pathogenesis of Koala Retrovirus
Source: Viruses. 2025 Nov 17;17(11):1510. doi: 10.3390/v17111510 (PMC12656766; doi:10.3390/v17111510)
Supplement: Supplementary file 1 [file viruses-17-01510-s001.zip › viruses-3911637-Supplementary Figures.pdf]

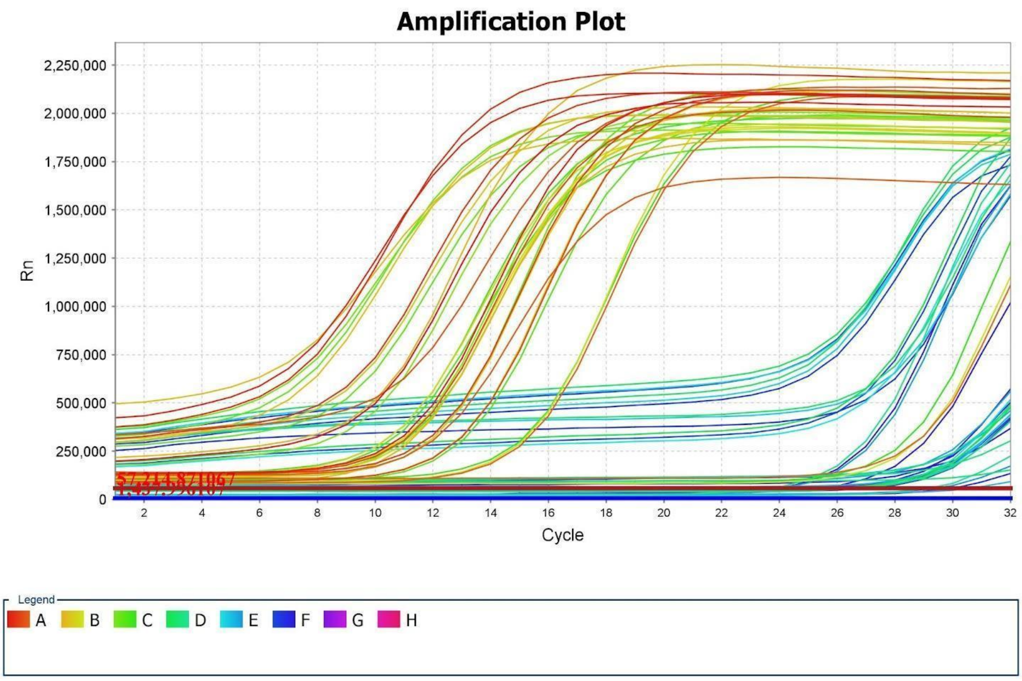

Figure S1: Amplification plot showing successful qPCR amplification upon TEL and SCR primers in the first batch of the experiment containing 12 samples, including positive and negative controls;

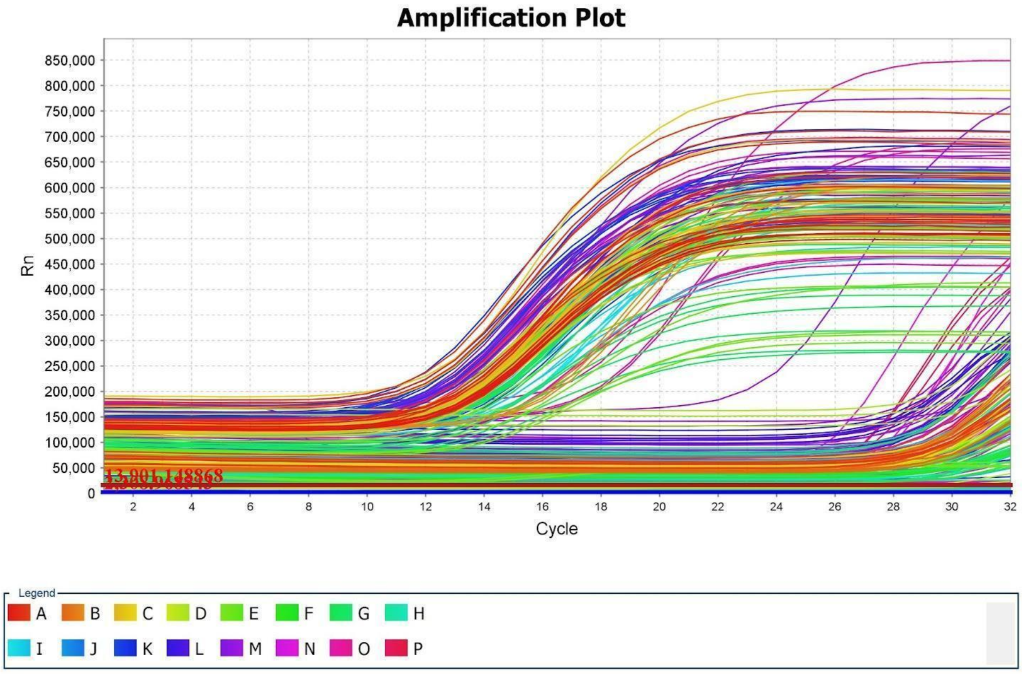

Figure S2: amplification plot showing successful qPCR amplification upon TEL and SCR primers in the second batch of the experiment containing 50 samples, including positive and negative controls;
